# Implementation of the Chick Chorioallantoic Membrane (CAM) Model in Radiation Biology and Experimental Radiation Oncology Research

**DOI:** 10.3390/cancers11101499

**Published:** 2019-10-07

**Authors:** Nicole Dünker, Verena Jendrossek

**Affiliations:** 1 Institute for Anatomy II, Department of Neuroanatomy, University of Duisburg-Essen, University Medicine Essen, 45122 Essen, Germany; 2 Institute of Cell Biology (Cancer Research), University of Duisburg-Essen, University Medicine Essen, 45122 Essen, Germany

**Keywords:** chorioallantoic membrane assay, CAM, radiation, radiotherapy, radioresistance, hypoxia, cancer, tumor, molecularly targeted drugs, tumor microenvironment

## Abstract

Radiotherapy (RT) is part of standard cancer treatment. Innovations in treatment planning and increased precision in dose delivery have significantly improved the therapeutic gain of radiotherapy but are reaching their limits due to biologic constraints. Thus, a better understanding of the complex local and systemic responses to RT and of the biological mechanisms causing treatment success or failure is required if we aim to define novel targets for biological therapy optimization. Moreover, optimal treatment schedules and prognostic biomarkers have to be defined for assigning patients to the best treatment option. The complexity of the tumor environment and of the radiation response requires extensive in vivo experiments for the validation of such treatments. So far in vivo investigations have mostly been performed in time- and cost-intensive murine models. Here we propose the implementation of the chick chorioallantoic membrane (CAM) model as a fast, cost-efficient model for semi high-throughput preclinical in vivo screening of the modulation of the radiation effects by molecularly targeted drugs. This review provides a comprehensive overview on the application spectrum, advantages and limitations of the CAM assay and summarizes current knowledge of its applicability for cancer research with special focus on research in radiation biology and experimental radiation oncology.

## 1. Introduction

About 50% of all cancer patients receive radiotherapy (RT) at some point during the course of their disease (World Health Organization, WHO) and good results in terms of long-term survival and tumor cure are achieved in a variety of tumors by multimodal combinations of surgery, RT, and chemotherapy [1]. Yet cure rates remain still unsatisfactory for common forms of cancer with high loco-regional failure-rates or frequent development of metastases. Though patient-specific clinical factors may explain some of these failures, it is commonly assumed that multiple biological factors adversely affect the response of tumor cells to treatment. Major biological limitations to successful RT comprise tumor-promoting mutations, unfavorable gene expression profiles, high intrinsic cancer cell radioresistance, a resistance-promoting microenvironment, hypoxia, heterogeneity in tumor and normal tissue radiation responses, phenotypic plasticity of cancer cells in adverse environments, tumor heterogeneity, as well as enrichment in radioresistant tumor stem cells [2]. Moreover, acute and late toxicity to normal tissues limit the radiation dose that can be safely applied to the tumor and decrease the quality of life of cancer patients, whereas tolerable doses are often linked to suboptimal tumor control [3,4,5,6,7]. These limitations highlight the high medical need for innovations in RT practice.

Innovative strategies for improving treatment outcome therefore aim to selectively enhance toxicity to the tumor, reduce complications in normal tissues, or both. During recent years, the practice of RT has substantially benefited from technical improvements in treatment planning and increased accuracy of dose delivery (e.g., stereotactic RT or intensity-modulated radiation therapy (IMRT)), as well as by the development of particle therapy approaches [8]. Charged particles (mainly protons and carbon ions) have depth-dose distributions and linear energy transfer (LET) characteristics that allow the deposition of high and more effective radiation doses to deep-seated regions in a patient [8,9,10,11].

Safety and effectiveness of therapy with protons or other charged particles are currently evaluated in clinical studies in specialized centers for carefully selected tumor sites [12,13,14]. For example, the advantageous physical and radiobiological characteristics of carbon ions make carbon ion therapy an attractive approach for the treatment of hypoxic tumors with pronounced radioresistance, e.g., non-small cell lung cancer [15,16,17]. Nevertheless, further preclinical work is required to define the relative biological effectiveness (RBE) of particle beams of different energies in clinically relevant tumor entities in vitro and in vivo. Furthermore, the increased RBE of carbon ion beams compared to photon and proton beams with respect to the eradication of clonogenic tumor cells and the reduction in tumor burden may also increase the risk for acute and chronic adverse effects in tissues and organs with high intrinsic radiosensitivity such as the normal lung tissue [18,19,20,21,22,23]. It is thus also important to evaluate the RBE of protons, carbon ions, and heavy ions with respect to sensitivity of cells from normal tissues in vitro as well as the tolerance of highly radiosensitive normal tissues and organs to such treatments in preclinical models in vivo [19,24,25]. Taken together, despite success, improving cure-rates through technical and physical improvements in accurate dose delivery has some natural limits defined by the biological characteristics of the tumor or the infiltration of critical normal-tissue structures by malignant cells that cannot be spared, further underlining the need for further innovation in radiotherapy practice.

## 2. Biologic Optimization of Radiotherapy

Another important approach for improving cure rates focuses on the biologic optimization of RT by targeted modulation of biological processes that determine the radiation response of normal and tumor tissues. A few successful examples document promise of such approaches: RT plus androgen deprivation to treat locally advanced prostate cancer or RT plus cetuximab, an inhibitory antibody against the epidermal growth factor receptor (EGFR), to treat head and neck cancer patients not tolerating concurrent cisplatin chemotherapy (CT) [26,27,28,29] and more recently, immunotherapy [30,31]. However, other clinical trials combining chemoradiotherapy (CRT) with small molecule signal transduction inhibitors did not yet translate into clinical practice [29] or have even been negative due to low efficacy or complications [32,33,34,35]. Thus, further preclinical work is required to gain a better understanding of the complex local and systemic responses to RT and their modulation by application of targeted drugs, of intra- and inter-cellular signaling networks that control cell radiosensitivity and cell fate decisions, and of the mechanisms driving microenvironment-mediated resistance if we aim to design effective strategies for radiation response modulation [3,36,37].

Improvements in the outcome of patients with locally advanced tumors are currently expected from treatment protocols combining precision RT using highly conformal photon radiotherapy or particle therapy with molecularly targeted small molecules targeting for example signaling pathways in tumor cells or environmental factors promoting cancer (stem) cell radioresistance, genetic vulnerabilities, cancer driving mutations, or other malignant cancer traits [25,37,38,39,40,41]. Herein, the identification of specific differences in radiation response pathways and genetic vulnerabilities between cancer and normal cells will offer unique opportunities for cancer cell-specific modulation of the radiation response.

DNA double strand breaks (DSB) are the most crucial lesions induced by ionizing radiation. Cells resist the toxic effects of ionizing radiation by mounting an efficient DNA damage response (DDR) [42] to regulate, adapt, and coordinate many vital cellular functions upon detection of DSBs, and to effectively repair radiation-induced lesions, particularly DSBs. Thus, compounds that suppress DDR or DSB repair have the potential for tumor radiosensitization, particularly when they affect tumor cells more than the surrounding normal tissue; vice-versa, compounds that selectively enhance DDR and DSB repair in normal cells may be used for normal tissue protection [3,43,44,45,46,47,48]. Currently, exciting potential for innovation arises from the identification of cancer cell-specific or context-dependent (e.g., severe hypoxia) deficiencies in DNA repair or genomic stability, that may be exploited for cancer cell-specific or tumor region-selective radiosensitization, respectively [45,49,50,51,52,53,54,55].

Of note, preclinical investigations revealed differences in the quality of DNA damage induced by irradiation with photons and charged heavy ions [56,57,58,59,60,61,62] and potentially even between photons and protons [11,63,64]. The enhanced requirement for specific DNA repair pathways, particularly homologous recombination repair (HRR), may have potential relevance for stratification of patients carrying mutations in DNA damage response or DNA repair pathways [63]. The assumed enhanced vulnerability of such cancer cells to proton or particle irradiation may open new avenues for optimization of particle therapy. However, extensive further preclinical work is required to further define the underlying mechanisms and to explore the potential of such differences for the design of rational and effective combinatorial approaches using proton therapy or therapy with other charged particles in combination with targeted DNA damage response modifiers. Successful individualization of RT quality and mechanism-based combinatorial strategies in the clinics will depend on the definition of reliable biomarkers or alternatively of gene-expression, miRNA-expression or protein-expression profiles predictive for individual tumor and normal tissue radiosensitivity; such profiles can be identified by systematic multiomics analysis of individual expression profiles in irradiated individual tumor and normal tissues of individual patients [39,40,65,66,67,68].

Another emerging concept for biology-based optimization of RT is based on the observation that the tumor stroma impacts cancer cell radiosensitivity at multiple levels and may thus provide attractive therapeutic targets. Herein, extracellular matrix (ECM) molecules, tumor microvessels, the vascular stem cell niche, accessory host cells (e.g., fibroblasts and cells of the innate and adaptive immune systems), and secreted factors constitute important determinants of the tumor response to RT, at least in preclinical investigations [69,70,71,72,73,74,75]. Although the first observations about a potential contribution of the host immune system to successful tumor eradication by the local application of ionizing radiation date more than 100 years ago (for review see: [76]), clinical observations about radiation-induced eradication of tumor lesions outside the radiation field (abscopal effects) remained rare [77]. The progress in immunotherapy and the seminal discoveries that radiotherapy can overcome immunosuppressive barriers in the tumor microenvironment, induce immunogenic alterations in tumor cells, and even elicit local and systemic T-cell-mediated antitumor immune responses expedited interest in exploiting the benefit of combining radiotherapy with immunotherapy [30,31,77,78,79,80,81,82,83,84,85,86,87,88,89,90,91]. This knowledge is now being increasingly used in the design of new treatment strategies, e.g., by combining RT with immunotherapy [84,92,93,94]. For example, first clinical studies revealed that lung cancer patients benefit from a treatment with inhibitors of the programmed cell death protein1 (PD1)/PD-1 ligand 1 (PDL1) immune checkpoint when given after RT or platinum-based CRT with improved progression-free survival and that this treatment had an acceptable safety profile [95,96]. However, a better understanding of the multifactorial mechanisms driving tumor immune escape, radiation-induced immune activation, and the reciprocal interactions between RT-induced and immunotherapy-induced immune changes is needed to optimally exploit RT-induced immune enhancement e.g., in combination with immune checkpoint inhibition, and to avoid the development of therapy resistance and excessive toxic side effects [97,98,99,100,101,102,103,104] and these investigations require in vivo models.

Finally, the microenvironment of solid human tumors is frequently characterized by reduced oxygen tensions including tumor types with pronounced radioresistance [105]. This so-called tumor hypoxia mostly coexists with a low tumor pH (tumor acidosis) further aggravating the hostile tumor environment [106]. Tumor hypoxia results from an imbalance between the pronounced oxygen demand by rapidly growing and proliferating cancer cells and an insufficient oxygen supply that is limited by irregular blood flow, a chaotic and dysfunctional tumor microvasculature, and potentially a reduced oxygen transport capacity and limited oxygen saturation in cancer patients [107,108,109]. Tumor hypoxia has prognostic relevance and is associated with poor patient outcome independent of their respective treatment [110]. However, except for heavy particle therapy with carbon ions tumor hypoxia is particularly relevant in radiation oncology as already highlighted more than 50 years ago [111] and confirmed by multiple reports about the prognostic value of pre-therapeutic tumor hypoxia to patient outcome upon RT [112,113,114]. Tumor hypoxia constitutes a major biological obstacle to successful RT for multiple reasons: On the one hand, the cytotoxic efficacy of RT relies on the formation of reactive oxygen species (ROS) and thus on local availability of molecular oxygen (O_2_) during treatment delivery. An acute decrease in O_2_ levels will thus result in reduced DNA damage and thus confer direct resistance to RT-induced cell killing [115,116,117]. These observations led to the concept of using of hyperbaric oxygenation [118], oxygen mimics, hypoxia-activated pro-dugs and hypoxia active drugs for radiosensitization as recently reviewed elsewhere [114]. Interestingly, pharmacologic approaches reducing oxygen consumption by metabolic inhibition turned out to be much more efficient in increasing oxygen availability in hypoxic tumors than approaches to increase oxygen delivery [119,120,121,122,123,124,125]. Furthermore, O_2_ deprivation restrains proliferation and activates drug-resistance genes as well as survival pathways that decrease therapy-induced cell death and promote treatment failure [126,127,128,129]; these observations make components of hypoxia-activated survival pathways promising therapeutic targets to overcome RT resistance in acute hypoxia [129,130]. However, oxygen availability in hypoxic tumor regions is not only highly heterogeneous ranging from mild (<5% O_2_) to severe hypoxia (<0.5% O_2_) or even anoxia (<0.01% O_2_) but also highly dynamic with respect to its duration and schedule (acute, transient, chronic, or intermittent) and also fluctuates regionally presumably as a result of the instability and chaotic organization of the tumor vasculature [109,131,132,133,134]. Substantial fractions of human solid tumors are thus exposed to dynamic cycles of hypoxia and intermittent re-oxygenation that have been demonstrated to drive adaptive responses and phenotypic heterogeneity as well as malignant progression by increasing genomic instability, generation and maintenance of cancer stem cells, cancer cell metastasis, and the clonal evolution of therapy-resistant cancer cells [37,130,131,135,136,137,138,139,140,141] and these processes are all highly relevant to cancer RT. Notably, further preclinical investigations revealed differences in the DNA repair proficiency between cells cultured under normal oxygen tensions compared to cells exposed to severely hypoxic conditions [54,55,142,143,144,145]. Furthermore, hypoxia is suspected to support certain aspects of tumor immune escape [146,147,148,149]. It is thus tempting to speculate that differences in DNA repair proficiency, the immune repertoire, or both might be exploited for a context-dependent radiosensitization of hypoxic tumors [150,151,152,153,154]. However, these innovative concepts will require extensive preclinical evaluation.

The progress in understanding the various molecular and cellular mechanisms underlying hypoxia-driven radiation resistance allowed for the development of innovative concepts for improving the RT response of solid tumors with substantial hypoxic fractions that warrant validation in independent preclinical studies and the clinical context.

Taken together, molecularly targeted drugs are expected to become part of future innovative RT and concurrent CRT protocols designed to treat solid tumors. Because of the broader availability of patient irradiation with photon beams so far, the in vitro and in vivo validation of such combinatorial approaches has been performed with photon irradiation. In the future, these biological approaches are likely to be combined with highly conformal RT and particle therapy, to harness the combined potential of all [155,156,157]. As mentioned above, successful clinical translation of such approaches will require the definition of reliable biomarkers or gene or protein expression signatures predictive for individual radiosensitivity of tumor and normal tissues and for responsiveness to specific therapies and diverse response modifiers [39,40,65,66,67,68,158]; such biomarkers tor radiosensitivitiy profiles will help to individualize RT dose prescriptions and the selection of appropriate combination strategies in the future.

## 3. Current Preclinical Models to Assess Cellular Radiation Responses

Preclinical in vitro investigations in translational cancer research mostly focus on established cancer cell lines (2D, 3D, co-culture systems) as well as patient-derived primary cells, tissue slices (organotypic cultures), or organoid cultures derived from normal and cancer tissues, if available [158,159,160,161,162,163,164,165]. Organotypic tissue slice approaches offer the advantage to study effects in a more physiological cell–cell network context, retaining the native multicellularity, architecture, heterogeneity and physiology of the complex tumor-stroma interactions in the natural tumor microenvironment, at least in short-term cultures [166]. But the method requires access to patient samples, extensive experience and laborious optimization of culture conditions for extended culturing times [167], for avoiding tissue hypoxia, and for establishing appropriate clinically relevant end-points (for a comprehensive overview please refer to [168]). Once established, results with organotypic tissue slice cultures can be obtained within days suggesting that this method may be well suited for patient stratification in personalized medicine [168,169]. Though established from dissociated normal tissues or isolated cancer stem cells, organoids also comprise heterogeneous cell subtypes and their response to experimental treatments still more closely resembles the in vivo situation than cell lines. Besides, organoids with their cell–cell and cell-matrix interactions and the capacity of cellular differentiation offer a much higher complexity than traditional 2D cell systems [170]. However, the vascularization of a “natural” tumor and also its microenvironment influence cancer treatment responses and cannot easily be phenocopied neither by tissue slice cultures nor by organoids, although recent advances in organ-on-a-chip methods address this issue by connected chamber constructions mimicking, e.g., blood vasculature [171]. Besides, next to their genomic instability another major drawback of organoids is the limitation of available tissue entities (for review see: [172]). Moreover, current challenges of organoid research are on the one hand to increase organoid efficiency and to decrease the time for organoid outgrowth and on the other hand to lower the costs, and develop high-throughput screening [165].

The above in vitro models are complemented by classical xenograft or orthotopic murine tumor models with established human or syngeneic murine cancer cell lines, genetically engineered murine tumor models, or patient-derived xenograft tumors grown in immunodeficient mice [173,174,175,176,177,178,179,180,181,182,183,184].

So far, the “gold-standard” for the determination of cellular radiation sensitivity in radiobiology investigations has been the evaluation of clonogenic cell survival [37]. However, researchers in experimental radiation biology aim to develop alternative approaches [185] and at integrating improved models for designing their preclinical investigations as close as possible to the clinical situation, e.g., when performing mechanistic studies, to define therapeutic targets, or to evaluate novel combinatorial treatment strategies. However, each model system has its inherent advantages and disadvantages to answer specific research questions. Moreover, some of the above models require highly specialized knowledge, expensive specialized instrumentation, and/or access to patient material. These resources may only be available at larger cancer research centers or comprehensive cancer centers. The use of these model systems in translational cancer research as well as the inherent advantages and disadvantages have been recently reviewed elsewhere with a special focus on patient-derived organoid cultures, and will therefore not be discussed in detail here [170].

What has been neglected in many RT studies and cannot be addressed in almost all in vitro systems (except for tissue slice and organoid cultures), is that in addition to tumor cells, the tumor comprises also stroma cells, i.e., fibroblasts, endothelial cells, and cells of the immune system, embedded in tumor-specific extracellular matrix [186]. In recent years, it has become evident that stroma is not a passive scaffold-component of the tumor. Rather, stroma actively influences tumor growth and presents an important determinant of its responses to therapeutic interventions. Stroma regulates several aspects of tumor growth and maintenance, such the supply of oxygen and nutrients through the tumor vasculature, the signal input by growth factors and ECM molecules, and even the tumor-associated inflammation [187]. Vice-versa, tumor stroma is necessarily irradiated together with tumor cells and elicits local and systemic responses, likely to modify the overall tumor response, both locally and systemically [188,189,190,191,192]. The impact of vasculature [75,193,194,195], extracellular matrix [72,196,197], and recruited immune cells [90,91,198,199] on RT response is actively debated and studied, and is thought to offer potential for innovation in the discovery of tumor radiosensitization targets. But further studies are required to enhance our understanding of the mechanisms regulating the interplay of tumor cells, stroma cells, ECM molecules, and recruited immune cells in tumor responses to RT.

## 4. The Chick Chorioallantoic Membrane (CAM) Assay

**Figure 1:**
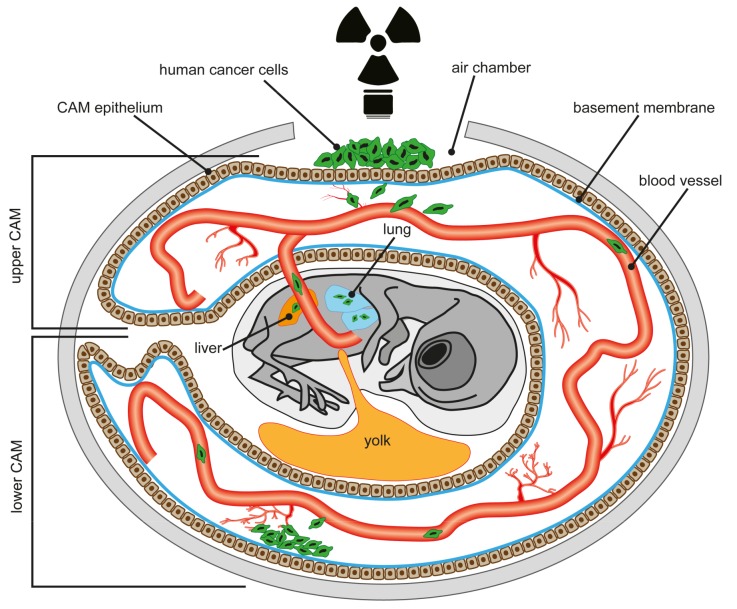
Schematic overview of the chorioallantoic membrane (CAM) assay. Modified after [201].

Between incubation day 4 and 10, the allantoic vesicle of the chick embryo enlarges and fuses with the adjacent mesodermal layer of the chorion to form the chorioallantoic membrane (CAM), which is highly vascularized and connected to the embryonic circulation by two arteries and one vein [200,201,202,203]. On gestation day 12, the CAM epithelium, physiologically serving as the chick embryo’s lungs, surrounds the whole embryo (see Figure 1). Histologically, the CAM contains three major layers: (1) the ectoderm, which is attached to the shell membrane; (2) the mesoderm comprising a rich vascularization; and (3) the endoderm facing the allantoic cavity [204,205]. On gestation day 10, the CAM ectoderm capillary plexus connecting the arterial and venous blood vessels is fully developed. The vasculature not only plays an important role in chick embryo development, but using the CAM as a tumor model excessive vascularization also impacts inflammation and tumor growth. As a prerequisite for therapeutic intervention, a study by Soulet et al. mapped the molecular repertoire of the extracellular and membrane proteome associated with the vasculature and stroma [206]. In this context, most recently Mangir et al. reported on the use of the CAM assay to visualize tumor induced changes in vasculature [207].

For CAM assays, fertilized eggs are incubated in a humidified rotary incubator at 38 °C and 50% humidity. Between embryonic (E) day 8, or preferentially E10, when the CAM vasculature is readily visible, the eggs are candled by shining light into the eggshell at the blunt end of the egg to locate the chorioallantoic vein, which is marked approximately 1 cm away from its branching point [208,209]. Afterwards, a hole is drilled through the blunt end of the egg into the air sac and the “upper CAM” is dropped by gentle suction, creating an artificial air chamber (see Figure 1). Another hole is drilled within the drawn square on the eggshell, the exposed CAM is gently abraded, e.g., by the use of a cotton-tipped applicator, and a suspension of cells or tissue biopsies can be placed onto the CAM. Then the window in the eggshell is sealed tightly with tape and the egg is returned to the incubator

Already in 1958, human squamous cell carcinoma, sarcoma, adenocarcinoma, and bronchogenic carcinoma samples have been implanted onto the CAM, evaluated for the presence of metastases in the chick embryo and the efficiency chemotherapeutics on their growth [210]. Kaufman et al. (1956) were the firsts to describe changes in the CAM adjacent to the tumor inoculation site [211]. Dagg et al. (1956) used the CAM assay to investigate the metastasis of human squamous cell carcinoma, sarcoma, and embryonal rhabdomyosarcoma on the CAM but also in the chick embryo [212]. Till then human tissue allo- and xenograft, e.g., from normal and cancerous bladder, liver, endometrium, adrenal gland, cerebellum, and skin have been successfully grafted onto the CAM by us and others and re-vascularize by developing anastomoses between donor and chick host vessels [213,214,215]. Besides, studies consolidated the CAM as a useful model to study metastasis [216,217].

The rapid development, tissue composition and the accessibility of the chick chorioallantoic membrane (CAM) for experimental manipulation provides an exceptionally useful preclinical in vivo model for scientists in different fields of research like bioengineering, biochemistry, transplant biology, cancer research, and drug development [218,219]. As the CAM is connected to the chick embryo by a rich vasculatory network, this circulatory system is easily accessible for observations and manipulations. Besides, the CAM allows for real-time observations of treatment-induced changes in tumor cells in vivo [204]. Next to its well-known role as an angio- or anti-angiogenesis model (for review see: [200,202,220]), the CAM model reliably simulates key features of tumor growth in a few days and thus significantly speeds up research on human tumor progression and preclinical drug screening [219]. The CAM assay can be used for the evaluation of the activity and toxicity of a drug in the CAM as well as in grafted tumors or tumors forming from inoculated cells and also on the developing chick embryo itself, e.g., in terms of embryo death, inflammatory, and neo-vascularizing effects. A most recent review by DeBord et al. propagates the CAM as an increasingly valuable platform for patient-derived xenografts in preclinical oncology research [221]. As the CAM responds to injury with an inflammatory process similar to that in rabbit eye’s conjunctival tissue, when it comes to testing for the irritancy potential of new chemicals, the CAM is likewise an in vitro alternative to the in vivo Draize rabbit eye test [222].

The nourishing vascularized structure of the CAM, enabling rapid tumor growth after inoculation of cells, also serves as an excellent metastasis model [205,208,209,223]. The capillary network of the CAM provides a repository for tumor cells disseminating from the primary site of grafting and intravasating into the hosts vasculature. For this purpose, fluorescent labeled, e.g., green-fluorescent protein (GFP)-labeled human cancer cells are inoculated on the upper CAM (see Figure 1). Depending on their tumerigenic potential the cells will eventually invade the upper CAM epithelium and basement membrane to enter a blood vessel. Highly aggressive cells thereby metastasize to the lower CAM, where they evade from the vasculature or invade the liver or lung of the chick embryo. Thus, in this setting the CAM provides an in vivo model to study the intravasation step of the metastatic cascade. The duration of the CAM assay is limited to a 7–9-day window available before the chick hatches. Tumors which form from the grafted cells are excised, measured, and photographed [223,224,225].

In the experimental migration setting, tumor cells are injected into a large CAM vein and the capillary system provides a site for initial arrest, but later on tumor cells may extravasate into the surrounding tissue [208,226]. After distinct time points standardized tissue punches of the lower CAM are collected, scanned for GFP-positive retinoblastoma RB cells by fluorescence microscopy, and collected in buffer or cryoconserved for RNA extraction. Intravasation, vascular dissemination, and migration of human tumor cells in the chick embryo’s membranes and tissues can be quantified by flow cytometry analysis using a fluorescence –activated cell sorter (FACS, PCR mediated amplification of human-specific *Alu* sequences [208], or real-time PCR using human-specific primers, e.g., for glycerinaldehyd-3-phosphat-dehydrogenase (GAPDH) [223,224,227].

On incubation day 3 or 4 the embryo and its extraembryonic membranes can also be transferred to a Petri dish and further incubated as an ex ovo culture [203,213,228]. This system improves the accessibility of the embryo and CAM tissue and facilitates live imaging and microsurgery applications [229,230]. Besides, this method allows for the analysis of several samples in one setting as well as the quantification of treatment responses over a larger CAM area. Ex ovo CAM experiments with chick embryos grown in a shell-less environment provide an ideal set up for high-resolution fluorescent microscopy approaches as the fluorescent-labeled chicken vasculature as well as labeled cancer cells can be visualized simultaneously; thus, all steps of the metastatic cancer cascade including cancer cell migration and finally extravasation from the blood vessels can not only be imaged intravitally but also quantified using proper image analysis tools [231,232].

## 5. Advantages and Limitations of the CAM Assay

The CAM offers a plethora of advantages over the standard murine model to study, for example, therapy effects on tumor growth, the multistep process of tumor metastasis, angiogenesis, and drug sensitivity [233]. The CAM is naturally at least partially immune-deficient and thus, able to receive xenotransplants from various tissues and species without specific or nonspecific immune response. Another advantage is that the rich blood vessel and capillary vascular network of the CAM, naturally providing an interface for gas exchange for the chick embryo, also allows for survival, growth, and rapid vascularization of CAM tumors formed by inoculated cancer cells, but also bioptic patients’ tissue specimens implanted on the CAM surface (for review see: [203,204,205,221,234,235,236]).

### 5.1. Biological Advantages

Compared to costly and time consuming rodent animal models, where tumor growth often takes up to 6 weeks, the CAM is an inexpensive, experimentally easily accessible, and quick model, in which tumors become visible between 2 and 5 days after tumor cell inoculation. Thus, the CAM allows for high-throughput screening of large sample numbers [203]. Another advantage of the CAM model is that cancer cells arrested in the CAM microcirculation survive and a large number extravasate while in standard rodent models intravenously injected tumor cells often rapidly perish before extravasation. Besides, labeled human tumor cells can not only be identified in the CAM, but also in the chick organs [237]. Strikingly, Chambers et al. described a higher number of tumors and metastases in a larger variety of organs after intravenous tumor cell injection in the chick vs. the murine model [217].

Although the duration of the CAM assay is limited to a time window before chick embryo hatching, CAM tumors developing from implanted cells or tissue biopsies have been successfully re-grafted by us [213] and others (for review see: [204,238,239]) prolonging the experimental time frame. Nonspecific inflammation, another limitation of the CAM model, can at least be partially prevented by an early onset of the implantation procedure (around day 9), when the chick’s immune system is still completely immature.

### 5.2. Technical Advantages

Besides, while radiosensitizing experiments in mice require fixation of the animals under anesthesia, chick embryos can be easily irradiated without the need of any intricate operation [240].

### 5.3. Ethical Advantages

An ethical advantage of the CAM assay is that the CAM itself is not innervated and experiments are terminated before the development of centers in the brain associated with pain perception [203]. Thus, the chick embryo is not considered a living animal until E17 (in most countries) or even until hatching. At least in Germany the CAM assay is not classified as an animal experiment under the guidelines for handling laboratory animals, can be used without any ethical restriction and does not require protocol approval by an animal welfare or ethics committee. Table 1 provides a summarized overview of the main advantages of the CAM model compared to murine cancer models.

## 6. The Immune System of the CAM

Tumors have been compared to a complex ecosystem as the cancer cells themselves are surrounded by a highly dynamic microenvironment consisting of extracellular matrix, and diverse cancer-associated cells, involved in the modulation of cancer cell activity and tumor progression [241,242]. While around a dozen successful patient-derived xenograft tissues of different tumor subtypes have been reported (for review see: [221]) and thus, the current literature strongly supports the CAM´s potential as an efficient preclinical in vivo model, its use would highly benefit from a better understanding of the interaction of the CAM xenograft with the developing chick-host immune system. Inflammatory cells represent an important component of the tumor microenvironment. The production of T and B lymphocytes in the CAM starts around day 11, but immune cells are not fully mature before the chick embryo hatches at day 21 [243]. In day 10–15 chick embryos heterophils, the chick homolog of mammalian neutrophils, and monocytes are the two major inflammatory cell types present [205]. Klingenberg et al. described macrophages, lymphocytes, and heterophilic granulocytes to be the most abundant immune cells in CAM tumors formed from xenografted Burkitt lymphoma BL2B95 cells [244]. The authors propagate the applicability of the CAM model for research with a focus on tumor–stroma interaction [244]. A more recent study showing that MCF-7 breast cancer cells turn the CAM into a tumor stroma surrogate supports this notion [245].

## 7. Radiation Studies in and Ex Ovo

So far, only a limited number of research groups in the field of radiobiology make use of the CAM model to investigate the impact of ionizing radiation on tumor growth and therapy response as well as angiogenesis. Importantly, first reports reveal the successful implementation of the CAM as a preclinical xenograft tumor model to study different schedules of ionizing radiation and drug dose reduction options, providing leads for the optimization of combined CRT [246]. Along this line, Kauffmann et al. used the ex ovo CAM model to investigate the effects of 2.5–10 Gy irradiation on sliced patients’ squamous cell carcinoma tumor specimen [229] as this system facilitates the access of tumor specimen not only for pharmaceutical applications but also for focused radiotherapy. Another study used the ex ovo chicken CAM to evaluate the effects of radiation combined with SPARC (secreted protein acidic and rich in cysteine) overexpression in neuroblastoma cells and describes a SPARC-induced reduction in radiation-induced angiogenesis [247]. More recently, another CAM study revealed a decrease in colorectal carcinoma growth and radioresistance upon depletion of hnRNP K (heterogeneous nuclear ribonucleoprotein K) [248].

The CAM model has also been used as a bioassay to evaluate the potential of a variety of photosensitizers [249,250] and an in vivo model for photodynamic therapy or two-photon excitation [251,252] and is now propagated as an alternative to the mouse tumor model in the attempt to assay tumor response to photodynamic therapy [253].

The majority of research on the biological effects of ionizing radiation focused on its impact on DNA damage and cytotoxicity. Comparatively little is, however, known about radiation’s effect on the microenvironment. For a long time, studies on the function of microenvironment on tumor progression have been hampered by the lack of an appropriate in vivo model till studies showed that the CAM is a highly suitable system for studying the interaction of tumors with the surrounding stroma [254,255].

When a tumor is subjected to radiotherapy, not only the cancer itself, but also the host cells are exposed to ionizing radiation. Macrophages, one major population of cells associated with tumors’ innate immune response, have been shown to play an important role in tumor progression and modulating therapy response [256]. Since macrophages have been shown to induce angiogenesis via secreted pro-angiogenic cytokines and chemokines, Pinto et al. made use of the CAM assay to evaluate the angiogenic potential of conditioned medium from irradiated and non-irradiated macrophage-colorectal cancer co-cultures [256].

Growing tumors compensate for their constantly increasing oxygen consumption by angiogenesis and vasculogenesis. Thus, one major goal in fighting against cancer is the inhibition of angiogenesis. In this context, the effects of irradiation and a static magnetic field on tumor-induced angiogenesis was tested in CAM assays [257,258]. Moreover, a study by Brooks et al. demonstrated that at 20 cGy new blood vessel formation in the CAM is inhibited by 85%–90% [259]. Besides, the CAM model has been used to study the effects of X-rays—alone or in combination with substances like nitric oxide—on angiogenesis [260]. In 2001 Karnabatidis and colleagues evaluated the effect of ionizing radiation on the angiogenesis process in the CAM model [261]. A study on the effects of combining ionizing radiation with drug treatment did not reveal any anti-angiogenic effect of the drug, but further confirmed the notion that the CAM is a convenient model for radiobiological studies [262].

Thus, within a certain time window the CAM offers radiobiologist a real-time follow-up on RT and CRT induced changes in tumor size and (neo-)angiogenesis as well as early metastasis in situ, which can easily be documented by imaging. Besides, it serves an excellent model to study RT effects on tumor cell proliferation and apoptosis by excising CAM tumors developing from grafted tumor cells or patients’ tumor biopsies after different radiation schedules or following different doses of CRT ex vivo and measuring cell viability, proliferation, and death via respective assays (e.g., WST-1, (TUNEL (terminal deoxynucleotidyl transferase dUTP nick end labeling assay), flow cytometry) in homogenates or immunohistochemically in histological sections. Moreover, radiation induced DNA damage and repair mechanisms and the effectiveness of radiosensitizing compounds suppressing DDR or DSB repair can be analyzed via PCR, electrophoresis, or immunohistochemically in samples from excised CAM tumors derived from inoculated tumor cells or tumor biopsies. A major advantage herein is the opportunity to perform such in vivo investigations in proton or heavy ion therapy centers that do not have opportunities for irradiation of small animals.

## 8. The CAM Model in Invasion Studies Under Hypoxia

The CAM model can also be used to study the effects of a hypoxic tumor microenvironment on tumor cell response to therapy, dissemination, and invasion. It has been shown that inoculated SW-490 colon carcinoma cells did not invade the epithelial layer of the CAM under normoxic conditions, but did under hypoxic conditions, indicating that hypoxia generates a more invasive phenotype of these tumor cells and establishing the CAM model for tumor invasion study under defined hypoxic conditions [263]. Along this line, Sun et al. employed the CAM invasion assays to show that hypoxia promotes ovarian cancer cell invasion via Snail-mediated upregulation of membrane-type 1 matrix metalloproteinase [264]. Another study used the CAM assay to characterize the effects of hypoxia-inducible factor-1 alpha (HIF-1), which is highly expressed under hypoxic conditions and commonly activated in tumors, especially in highly invasive and thus, aggressive tumors, on the angiogenic potential of small cell lung carcinoma cells [265]. In U87 glioma cell nodules, grafted on the CAM, the synthetic compound *myo*-inositol tri-pyrophosphate (ITPP), which increases the partial oxygen pressure (pO_2_) in hypoxic tissues, markedly reduced tumor progression and angiogenesis [266].

A more recent study investigated the influence of hypoxia on disseminating human head and neck squamous cell carcinoma cell fate in ovo. In the study setting, a hypoxic microenvironment generated by a nano-intravital device (NANIVID) induced a subpopulation of dormant, post-hypoxic disseminating tumor cells, which evaded chemotherapy and might thus be the source of disease relapses [267].

Importantly, since tumor hypoxia is an important biologic constraint to the efficacy of radiotherapy, the CAM model may offer the opportunity to perform investigations about the effects of hypoxia on the radiotherapy response and combinatorial treatments to overcome hypoxia-induced radioresistance. In fact, Abe et al. demonstrated that adjunction of 8 Gy irradiation and application of 1 mg etanidazole, a hypoxic cell radiosensitizer, significantly decreases the size of CAM tumors forming after inoculation of EMT6/KU murine mammary cells [240].

## 9. Conclusions

Molecularly targeted drugs are expected to become part of future innovative complex CRT protocols including either photons or particle irradiation designed to treat solid tumors. On the basis of preclinical studies describing the CAM as a tumor stroma surrogate and propagating its applicability for experiments on tumor-stroma interaction and combinatorial treatments, we propose the CAM as a useful preclinical model for in vivo investigations of hypothesized synergistic effects of radiation–drug combinations. The implementation of the CAM assay in radiation research would allow for a time- and cost-limiting reduction of classical experimental animal models and for an ideal complementation of investigations with tumor organoids and organotypic tissue slice cultures, respectively. While CAM tumors developing from inoculated human cells are surrounded by chick tissue, organoids are only composed of one type of human cells, a fact that might prove advantageous for subsequent functional analyses. However, the embryonic microenvironment of the chick CAM provides the opportunity to additionally study its influence on tumor growth and metastatic progression. Another advantage of the CAM over organotypic tissue slice cultures and organoid models is that its rich capillary vascular network not only allows for growth, survival, and rapid vascularization of tumors developing from inoculated tumor cells without limitations, e.g., in oxygen availability of organotypic tissue slice cultures and of organoids, but also nourishes patient-derived xenografts, allowing for long-term growth and expansion. Furthermore, the blood vessel network of the CAM provides a repository for tumor cells disseminating from the primary site of grafting, thus allowing for fast invasion and metastasis studies. Besides, as developing and expanding CAM tumors are connected to the chick vasculature, on the one hand RT effects on vascular remodeling and (neo-)angiogenesis, on the other hand the impact of the vasculature on RT responses can be studied.

We assume that the CAM model may also be an ideal complementary preclinical in vivo model to study the relative biological effectiveness (RBE) of radiotherapy with photons compared to particle beams of different energies in clinically relevant tumor entities and to compare the radiosensitivity of xenografted normal and tumor tissues. Although the CAM assay proved to be an extremely useful supplementary tool for preclinical studies, thus possibly reducing animal studies, it will not be able to completely replace them as some limitations have to be taken into consideration. The duration of the CAM assay is limited to a maximum of 7 days, restricting the time for more complex studies involving sequential treatment or fractionated radiation schedules. Furthermore, the metabolism of drugs will probably not be reflected accurately in the CAM assay, potentially limiting its value as a preclinical assay in this respect. Finally, as the immune system of the developing chicken is not fully mature and thus, does not resemble the adult human immune system, this aspect may limit application of the assay for immunotherapy-related studies. Nevertheless, the CAM assay will allow for high flexibility regarding the experimental design, e.g., screening of several molecularly targeted drugs, use of different radiation schedules and qualities, comparison of normal and tumor tissue responses, evaluation of the impact of hypoxia, and potential analyses of mechanisms regulating recruited immune cells in tumor response to RT including vascular immune effects. At the same time the CAM assay contributes to the ethical 3R (replacement, reduction, refinement) principle for the use of animals in research, although the presence of a developing chicken embryo may raise ethical issues as well.

## Figures and Tables

**Table 1:** Overview of advantages and limitations of the chick chorioallantoic membrane (CAM) model.

Issue	Studies in Mice	CAM Assay	Advantages CAM	Limitations CAM
Duration	4–9 (-12) weeks	3–5, max. 7 days	High throughput	Limited time frame for tumor growth and effects
Experimental burden	Middle to high burden due to invasive treatment and tumor growth	No to low burden due to mainly extraembryonic tumor development	Meeting the 3R principle	
Costs	High expenses for breeding, keeping, feeding	Low expenses for eggs and transport	Cost-saving (approx. 60% saving of expenses)	
Space requirements	High; specific condition required	Low	Space-saving	
Permission requirements	Protocol approval by animal welfare and ethics committee	No approval by welfare or ethics committee required *	No administrative burden; quicker study start	
Functional analyses	Availability of antibodies, cytokines, primers			Limited number of avian- compatible antibodies, cytokines, primers

* applies for Germany.
